# Targeting Secreted Protease/Anti-Protease Balance as a Vaccine Strategy against the Helminth *Fasciola hepatica*

**DOI:** 10.3390/vaccines10020155

**Published:** 2022-01-20

**Authors:** Krystyna Cwiklinski, Orla Drysdale, Jesús López Corrales, Yolanda Corripio-Miyar, Carolina De Marco Verissimo, Heather Jewhurst, David Smith, Richard Lalor, Tom N. McNeilly, John P. Dalton

**Affiliations:** 1 Molecular Parasitology Laboratory, Centre for One Health, Ryan Institute, National University of Ireland Galway, H91 DK59 Galway, Ireland; jesus.lopez@nuigalway.ie (J.L.C.); carolina.verissimo@nuigalway.ie (C.D.M.V.); heather.jewhurst@nuigalway.ie (H.J.); richard.lalor@nuigalway.ie (R.L.); johnpius.dalton@nuigalway.ie (J.P.D.); 2 School of Biological Sciences, Medical Biology Centre, Queen’s University Belfast, Belfast BT9 5DL, UK; o.drysdale@qub.ac.uk (O.D.); d.smith@moredun.ac.uk (D.S.); 3 Moredun Research Institute, Pentland Science Park, Penicuik, Midlothian EH26 0PZ, UK; yolanda.corripio-miyar@moredun.ac.uk (Y.C.-M.); tom.mcneilly@moredun.ac.uk (T.N.M.)

**Keywords:** *Fasciola hepatica*, sheep, vaccines, protease-anti-protease balance, stefin, Kunitz-type inhibitor

## Abstract

The liver fluke *Fasciola hepatica* is an economically important global pathogen of humans and their livestock. To facilitate host invasion and migration, *F. hepatica* secretes an abundance of cathepsin peptidases but prevents excessive damage to both parasite and host tissues by co-secreting regulatory peptidase inhibitors, cystatins/stefins and Kunitz-type inhibitors. Here, we report a vaccine strategy aimed at disrupting the parasite’s protease/anti-protease balance by targeting these key inhibitors. Our vaccine cocktail containing three recombinant stefins (rFhStf-1, rFhStf-2, rFhStf-3) and a Kunitz-type inhibitor (rFhKT1) formulated in adjuvant Montanide 61VG was assessed in two independent sheep trials. While fluke burden was not reduced in either trial, in Trial 1 the vaccinated animals showed significantly greater weight gain (*p* < 0.05) relative to the non-vaccinated control group. In both trials we observed a significant reduction in egg viability (36–42%). Multivariate regression analyses showed vaccination and increased levels of IgG2 antibodies specific for the *F. hepatica* peptidase inhibitors were positive indicators for increased weight gain and levels of haemoglobin within the normal range at 16 weeks post-infection (wpi; *p* < 0.05). These studies point to the potential of targeting peptidase inhibitors as vaccine cocktails for fasciolosis control in sheep.

## 1. Introduction

Helminth (worm) parasites have a major impact on global animal production, accounting for greater than 55% of all livestock disease [1,2]. One of the most prevalent diseases contributing to the negative impact on sheep and cattle production is caused by the trematode liver fluke parasite, *Fasciola hepatica*. Fasciolosis, the disease resulting from infection by liver fluke parasites results in major losses of €2.5 billion globally [3]. In Ireland alone, where the agri-food industry plays a vital economic role, it accounts for economic losses of €90 million per year [4]. As well as causing mortality, liver fluke causes subclinical production losses, with a recent meta-analysis estimating that liver fluke infections result in a 9% reduction in daily weight gain [5].

Parasite control is currently reliant on the use of anthelmintic chemicals, with the use of the benzimidazole class drug triclabendazole predominating for anti-fluke treatment [6]. However, within 15 years of its introduction in the early 1980s, widespread resistance was reported to this drug, compromising its future use [7,8]. The development of alternative sustainable and greener control strategies is now required for the long-term control of liver fluke. Vaccines are one approach currently being explored as a means to move away from chemical treatments [9,10].

Over the past decade, significant advances have been made in our understanding of liver fluke biology through the availability of large gene sequence datasets, specifically regarding the parasite stages involved in invasion of the host and establishment of the early stages of infection [11]. Focussing on two key life cycle stages involved in early infection, namely the newly excysted juveniles (NEJ) that migrate across the intestinal wall within 24 h of consumption of infectious metacercariae on pastures, and the damaging immature flukes that feed and migrate through liver tissue, we have identified several parasite molecules involved in *F. hepatica* pathogenesis and the immune suppression/modulation of host immune defences [12,13]. These molecules represent key targets at which vaccines could be developed to prevent critical parasite processes associated with the liver pathology, disease and loss of productivity observed during acute disease.

We have shown that the NEJ and immature fluke secrete an abundance of peptidases, including the cysteine peptidases cathepsin B3 (FhCB3) and cathepsins L (FhCL2 and FhCL3), that display collagenolytic activity, and play a major role in host penetration, parasite virulence, development, and suppression of immune responses [14,15,16,17]. The abundant expression of the cathepsin peptidases is consistent with previous studies that suggested that *F. hepatica* relies almost exclusively on this class of proteases [18,19,20]. Further interrogation of our secretome data revealed that two types of cathepsin inhibitors are also abundantly secreted during the early stages of infection, namely the cysteine protease inhibitors, cystatins also known as stefins and the Kunitz-type inhibitors [12,13,21]. The stefins and Kunitz-type inhibitors are potent inhibitors of the parasite cathepsin peptidases and play a key role in regulating the hydrolytic activity of these proteases [21,22,23].

In this study, we report a liver fluke vaccine strategy aimed at upsetting the intricate balance between peptidase activity and peptidase inhibition, also known as the protease/anti-protease balance, by targeting the parasite’s cathepsin inhibitors. Prevention of cathepsin protease regulation could alter the homeostasis of parasite-host interaction essential in tissue penetration, immune regulation and/or parasite feeding. Our recombinant vaccine cocktail was comprised of the major cathepsin inhibitors secreted by the NEJ and immature flukes, namely, three stefins (rFhStf-1, rFhStf-2, rFhStf-3) and a Kunitz-type inhibitor (rFhKT1). Vaccine efficacy was assessed in two independent livestock trials (sheep) which involved challenging animals with a pre-defined number of the infective metacercariae after vaccination.

## 2. Materials and Methods

### 2.1. Ethical Statements

All animal experimental procedures were carried out at Moredun Research Institute (MRI), UK under license from the Home Office by the Animal (Scientific Procedures) Act 1986 (License No. PPL/60/4426) after ethical review by the Moredun Animal Welfare and Ethical Review body.

### 2.2. Production of Functional Recombinant F. hepatica Stefins and Kunitz-Type Inhibitor

The FhStf-1 (BN1106_s4651B000094), FhStf-2 (BN1106_s2757B000215) and FhStf-3 (BN1106_s247B000268) sequences were derived from analysis of the *F. hepatica* genome (PRJEB6687) and were confirmed by the study by Cancela et al. [23]. The FhKT1 (BN1106_s8826B000029) sequence was previously described by Smith et al. [22]. Recombinant expression of the four *F. hepatica* antigens was carried out in the methylotrophic yeast *Pichia pastoris* with a C-terminal His-tag and purified using the protocol previously described [22,24]. Protein concentration and purity were verified by Bradford Protein Assay (Bio-Rad, Hercules, CA, USA) and by 4–20% SDS-PAGE gels (Bio-Rad, Hercules, CA, USA) stained with Biosafe Coomassie (Bio-Rad, Hercules, CA, USA), respectively. The gels were visualised using a G:BOX Chemi XRQ imager (Syngene, Cambridge, UK).

### 2.3. Whole Mount NEJ Immunolocalisation of FhStf-1, FhStf-2 and FhStf-3 by Confocal Microscopy

NEJ immunolocalisation studies were carried out as previously described [12] using excysted *F. hepatica* NEJ (Italian isolate; Ridgeway Research Ltd, St Briavels, UK) cultured for 6 h and 24 h that were fixed with 4% paraformaldehyde in 0.1 M PBS (Sigma-Aldrich, St Louis, MO, USA) for 1 h at room temperature. The anti-FhStf-1, anti-FhStf-2, anti-FhStf-3 or rabbit pre-immune antiserum were prepared in rabbits against recombinant FhStf-1, FhStf-2 and FhStf-3 (Eurogentec, Seraing, Belgium) using 200 µg of each antigen per immunisation; four injections in total were given at 0, 2, 4 and 8 weeks of the immunisation protocol. Cross-reactivity between the antibodies was assessed prior to immunolocalisation studies (Figure S1). The antibodies were used at 1:500 dilution. Bound antibody was visualised using a 1:200 dilution of the secondary antibody, fluorescein isothiocyanate (FITC)-labelled goat anti-rabbit IgG (Sigma-Aldrich, St Louis, MO, USA). Images were captured by confocal scanning laser microscopy (CSLM) (Leica TCS SP8, Leica Microsystems, Milton Keynes, UK) under the HCX PL APO CS 100x oil objective lens at room temperature and processed using the Leica Application Suite X (LAS X; v.2.0; Leica Microsystems, Milton Keynes, UK).

### 2.4. Inhibition of Cysteine Peptidase Activity in F. hepatica NEJ and Adult Secreted/Excreted Proteins (ES) by rFhStf-1, rFhStf-2 and rFhStf-3

The NEJ secreted ES proteins were recovered following 24 h culture (NEJ 24 h), as previously described [12]. Adult liver fluke parasites were recovered from experimental *F. hepatica* infections [25] and cultured for 5 h, as previously described [26,27,28].

The NEJ and adult ES proteins were incubated with the recombinant stefins, rFhStf-1, rFhStf-2 and rFhStf-3, at concentrations of 500 nM and 10 nM in a 100 µL volume reaction with 100 mM sodium acetate buffer pH 5.5 containing 1 mM DTT, 1 mM EDTA and 0.01% Brij L23 for 10 min at 37 °C. The reaction volume was then brought up to 200 µL with the fluorogenic substrates Z-Gly-Pro-Arg-NHMec (20 µM) for NEJ ES and Z-Leu-Arg-NHMec (20 µM) for adult ES dissolved in the sodium acetate buffer. Cysteine protease activity within the ES was measured for up to 1 h at 37 °C, as relative fluorescent units (RFU) using a PolarStar Omega Spectrophotometer (BMG LabTech, Aylesbury, UK). The broad-spectrum cysteine protease inhibitor E-64 (100 µM; Sigma Aldrich, St Louis, MO, USA) was used as a positive control. The inhibition of the recombinant *F. hepatica* peptidase inhibitors was calculated relative to the levels obtained with E-64, considered as 100%. All reactions were carried out in triplicate.

### 2.5. Vaccine Trial Design

Trial 1: Thirty-two 8-month-old Texel-cross sheep reared indoors at MRI, UK, to exclude accidental infection with helminth parasites (considered helminth-free) were allocated into three groups based on body weight. (G1) Group 1: control unvaccinated and uninfected (*n* = 4; 2 male, 2 female), (G2) Group 2: control unvaccinated and infected (*n* = 14; 2 male, 12 female), and (G3) Group 3: vaccinated and infected (*n* = 14; 3 male, 11 female) (Table S2). The number of animals per group was calculated using power analysis based on parasite burden at necropsy, assuming estimated infection rates in unvaccinated animals between 50 and 70% and average reduction in parasite number between 20 and 40% in vaccinated animals, as per other published studies in the literature [29].

Trial 2: Forty-seven 8-month-old male helminth-free Texel-cross sheep (MRI, UK) were allocated into four groups based on body weight. (G1) Group 1: control unvaccinated and uninfected (*n* = 8), (G2) Group 2: control unvaccinated and infected (*n* = 13), (G3) Group 3: vaccinated, infected (*n* = 13), and (G4) Group 4: vaccinated with the addition of CpG oligonucleotide and infected (*n* = 13) (Table S2). Sample size calculations were determined as above for Trial 1.

The vaccine trials were carried out unblinded. The vaccinated animals (Groups 3 and 4) received three subcutaneous injections, 3 weeks apart consisting of 400 µg (100 µg each of rFhStf-1, rFhStf-2, rFhStf-3 and rFhKT1) mixed in 2 mL Montanide 61VG (Seppic; ratio of adjuvant: antigen, 60:40). In Trial 2, 50 µg CpG (InvivoGen, Toulouse, France) was included in the vaccine preparation for Group 4. Three weeks after the second vaccination boost all animals, except for those in Group 1, were orally infected with 150 visually viable *F. hepatica* metacercariae in water (Trial 1: South Gloucester isolate; Ridgeway Research Ltd, St Briavels, UK; Trial 2: Italian isolate; Ridgeway Research Ltd, St Briavels, UK; based on the availability of triclabendazole susceptible *F. hepatica* isolates available at the time of the trials).

Body weight was assessed at the beginning of the study and prior to necropsy at 16 weeks post infection (wpi) for both trials. In Trial 2, body weight was also assessed at 4 wpi and 8 wpi. Faecal egg counts (FEC) were carried out using standard sedimentation protocols for the detection of *F. hepatica* eggs [25] at 12, 14 and 16 wpi. Animals were euthanised at 16 wpi (day 172/175 of the study) by lethal injection. At necropsy, the liver and gall bladder were recovered, and total enumeration of fluke burden and parasite eggs carried out. The size of all the adult flukes recovered was recorded (Trial 1: length and width; Trial 2: length and width, and estimates of fluke area).

### 2.6. Haematological Analysis and Liver Enzyme Assays

Blood samples were collected for haematological analysis and biochemical analysis of serum liver enzyme levels (glutamate dehydrogenase, GLDH, and gamma glutamyl transferase, GGT) by Scotland’s Rural College (SRUC Veterinary Services, Penicuik) at designated timepoints: (a) Trial 1: pre-challenge, 3 wpi and 16 wpi; (b) Trial 2: pre-challenge, 3 wpi, 8 wpi and 16 wpi. Haematological analysis was carried out to determine packed cell volume (PCV), red blood cell counts (RBC), total haemoglobin and differential white blood cell counts defined as a percentage of the total cell count for lymphocyte, neutrophil, eosinophil and monocyte subsets.

### 2.7. Egg Hatch Assay (EHA)

Eggs recovered from the gall bladder from each infected animal were washed in water and resuspended in 10 mL water. The eggs were diluted 1:100 and enumerated in triplicate in a volume of 100 µL. Embryonation and hatch rate were evaluated using standard *F. hepatica* egg hatch assay protocols [30]. Briefly, approximately 100 eggs from each animal were placed in duplicate, in a 12-well plate in 1 mL of tap water and embryonated in the dark at 26 °C for 14 days. The plates for embryonation assessment were exposed to light for 30 min prior to counting. The percentage of eggs at each developmental stage was determined by the protocol of Fairweather et al. [30]. Following the assessment of embryonation the 12-well plate was incubated overnight at 4 °C prior to egg hatch stimulation by incubation at 26 °C for 30 min followed by exposure to light for 2 h. A separate 12-well plate was set up to confirm the egg hatch rate. Following embryonation in the dark at 26 °C for 14 days, the plate was incubated overnight at 4 °C. Egg hatching was stimulated as detailed above. Egg viability based on hatch rate and percentage protection were calculated based on the average hatch rate from both the embryonation and hatch plates for the animals in each group, relative to the average egg hatch rates for the control infected group (G2).

### 2.8. Analysis of Anti-F. hepatica Antigen Specific Antibodies (Total IgG) in Sheep Sera Samples by ELISA

Flat-bottom 96 well microtitre plates (NuncTM Maxisorp TM, ThermoFisher Scientific, Roskilde, Denmark) were coated with 100 µL of 1 µg/mL of the recombinant antigens (rFhStf1, rFhStf2, rFhStf3, rFhKT1, or positive control rFhCL1) in 0.05 M carbonate buffer, pH 9.6 and incubated overnight at 4 °C. After five washes with 100 µL of PBS-0.05% Tween 20 (PBST), 100 µL/well of blocking buffer (2% bovine serum albumin diluted in PBST) was added and incubated for 1 h at 37 °C, followed by five washes with PBST. Serial dilution of the serum samples and the secondary antibody was carried out to determine the optimal concentration to be used in the assay. Sheep sera samples were diluted in serum dilution buffer (PBS, 0.5% Tween 80, 0.5 M NaCl) at 1:10,000 and 1:16,000, respectively for Trial 1 and Trial 2. One hundred µL of the diluted serum samples were added in triplicate to the plates and incubated for 1 h at 37 °C. After washing five times, 100 µL/well of HRP-conjugated donkey anti-sheep IgG (ThermoFisher Scientific, Carlsbad, CA, USA) diluted in blocking buffer (Trial 1, 1:30,000; Trial 2, 1:50,000) was added and the plates were incubated for 1 h at 37 °C. Following five washes, 100 µL/well of 3,3′,5,5′-Tetramethylbenzidine (TMB; Sigma-Aldrich, St Louis, MO, USA) was added and the plates were incubated at room temperature for 5.5 min. The reaction was stopped by the addition of 100 µL/well of 1 M sulphuric acid and the optical density was determined at a wavelength of 450 nm (OD450) in a PolarStar Omega spectrophotometer (Ortenberg, Germany).

### 2.9. Analysis of Anti-F. hepatica Antigen Specific IgG1 and IgG2 Isotypes in Sheep Sera Samples by ELISA

Flat-bottom 96 well microtitre plates (NuncTM Maxisorp TM, ThermoFisher Scientific, Roskilde, Denmark) were coated with 100 µL of 1 µg/mL of the recombinant antigens (rFhStf1, rFhStf2, rFhStf3, rFhKT1 or positive control rFhCL1) in 0.05 M carbonate buffer, pH 9.6 and incubated overnight at 4 °C. After five washes with 100 µL of PBST, 100 µL/well of blocking buffer (2% bovine serum albumin diluted in PBST) was added and incubated for 1 h at 37 °C, followed by five washes with PBST. Serial dilution of the sera samples and the secondary antibody was carried out to determine the optimal concentration to be used in the assay. Sheep sera samples were diluted in serum dilution buffer (PBS, 0.5% Tween 80, 0.5 M NaCl) at 1:1000 and 1:2000, respectively for Trial 1 and Trial 2. Fifty µL of the diluted serum samples were added in triplicate to the plates and incubated for 1 h at 37 °C. After washing five times, 100 µL/well of the anti-ovine IgG1 (McM1, ImmunoTools, GmbH, Friesoythe, Germany) or anti-ovine IgG2 (McM3, ImmunoTools, GmbH, Friesoythe, Germany) secondary antibodies diluted in dilution buffer at a concentration of 1:1000 and 1:2000, respectively, were added to the plates and incubated for 1 h at 37 °C. Following five washes, 50 µL/well of rabbit polyclonal anti-mouse Pan IgG-HRP (Dako, Glostrup, Denmark) diluted 1: 1000 in dilution buffer was added to the plates and incubated for 1 h at 37 °C. After washing five times, 100 µL/well of SIGMAFAST OPD (*o*-Phenylenediamine dihydrochloride; Sigma Aldrich, St Louis, MO, USA) was added and the plates were incubated at room temperature for 10 min. The reaction was stopped by the addition of 100 µL/well of 1 M sulphuric acid and the optical density was determined at a wavelength of 492 nm (OD492) in a PolarStar Omega spectrophotometer.

### 2.10. Isolation of Ovine Peripheral Blood Mononuclear Cell (PBMC) and Lymphocyte Stimulation Assays (LSA)

Blood was collected aseptically into sodium heparin vacutainers (Becton Dickinson, Oxford, UK) from eight representative animals per group from Trial 2 prior to challenge, and at 3 and 15 wpi. PBMC was isolated using density gradient centrifugation by layering whole blood diluted in PBS onto Ficoll-Paque™ PLUS (GE Healthcare Life Sciences, Chalfont St. Giles, UK). Buffy coat was collected, washed three times with PBS and re-suspended at 2 × 10^6^ cells/mL in RPMI-1640 medium supplemented with 10% heat inactivated foetal bovine serum (HiFBS; from USA supplied by Sigma-Aldrich, Dorset, UK), 50 µM 2-mercaptoethanol, 2mM L-glutamine, 100 U/mL penicillin and 100 µg/mL streptomycin.

Lymphocyte stimulation assays (LSA) were carried out with fresh PBMC at each sampling point to determine the specific proliferation induced by the recombinant *F. hepatica* protease inhibitors. Briefly, 2 × 10^5^ PBMC was stimulated in triplicate with equal volumes of PBS (negative control), Concanavalin A (Con A, positive T cell mitogen control, 5 μg/mL final concentration, Sigma Aldrich, St Louis, MO, USA) or individual heat-inactivated (1 h at 95 °C) *F. hepatica* antigens (rFhStf1, rFhStf2, rFhStf3 and rFhKT1), at 5 μg/mL final concentration, in a total volume of 200 μL, at 37 °C with 5% CO_2_ for 5 days. After 4 days, 50 µL of media from each replicate was discarded and replenished with RPMI-1640 media (as above) containing methyl-3H thymidine (0.5 μCi per well). Proliferation was measured by the incorporation of methyl-3H thymidine during the final 18 h of culture and expressed as stimulation index (S.I.) by dividing the corrected counts per minute (ccpm) obtained for samples stimulated with the *F. hepatica* antigens by those stimulated with PBS (negative controls).

### 2.11. Statistical Analysis

Statistical analysis was carried out using GraphPad Prism (v5.03, San Diego, CA, USA), unless otherwise stated. Group comparisons were assessed for normal distribution using the D’Agostino and Pearson omnibus normality test and the Shapiro Wilk normality test. Parametric data were analysed using one-way ANOVA with Tukey’s multiple comparisons (number and size of adult parasites recovered at necropsy, haematology data, log transformed liver enzyme data, ELISA data and PMBC stimulation data) and non-parametric data (number of eggs recovered from the gall bladder at necropsy to be used for the EHA and total weight gain) was assessed using the Kruskall–Wallis Test with Dunn’s multiple comparisons test. *p* values of <0.05 were considered significant.

Pairwise correlation analysis using Spearman’s rank correlation and multivariate regression analysis was carried out using R statistical software within R Studio (Version 1.4.11.03). For the multivariate regression analysis, all the numerical variables from the vaccine trial data were investigated for possible multicollinearity using pairwise correlation, and any variables displaying correlation coefficients of ≥0.8 were removed prior to regression analysis (Table S4). Weight gain, haemoglobin levels at 16 wpi and fluke number were used as dependent variables and remaining parameters were defined as independent variables. Essential variables/features for each model were determined using stepwise backward regression prior to analysis of multivariate regression.

## 3. Results

### 3.1. Recombinant Production of Functionally Active Proteins

#### 3.1.1. Recombinant Expression of Three *F. hepatica* Stefins and Kunitz-Type Inhibitor

**Figure 1:**
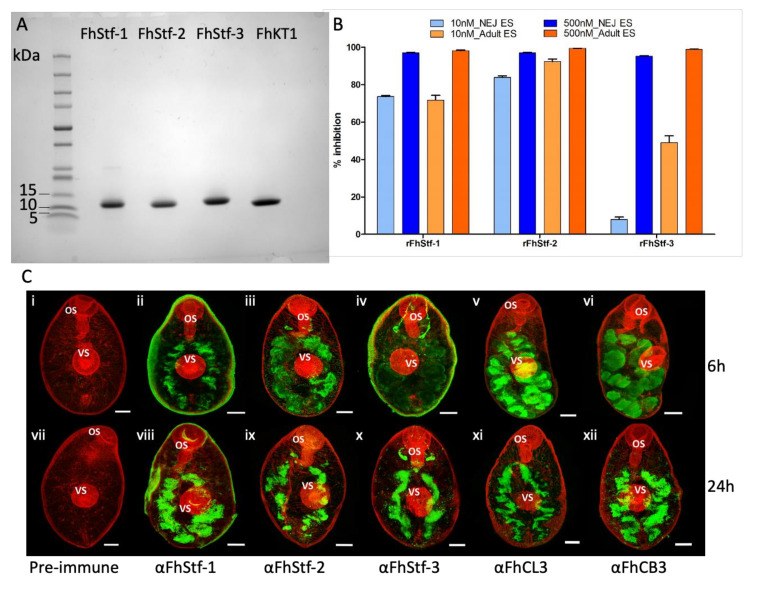
Characterisation of the *Fasciola hepatica* stefins. (**A**) Purification of the recombinant *F. hepatica* stefins and Kunitz-type inhibitors. 4–20% SDS-PAGE analysis of rFhStf-1 (lane 1), rFhStf-2 (lane 2) rFhStf-3 (lane 3) and rFhKT1 (lane 4). (**B**) Inhibition profiles of rFhStf-1, rFhStf-2 and rFhStf-3 against *F. hepatica* NEJ and adult ES products screened at 500 nM and 10 nM, relative to the cathepsin peptidase inhibitor E-64. E-64 inhibited the cathepsin peptidases within the ES products 100% (not shown on graph). Data represented at ± standard deviation of three replicates. (**C**) Immunolocalisation of FhStf-1, FhStf-2 and FhStf-3 in NEJ at 6 h and 24 h post-excystment by confocal laser microscopy. Following excystment, NEJ were maintained in culture and sample parasites taken at 6 h post-excystment (panels i–vi) and 24 h post-excystment (panels vii–xii). Parasites were probed with rabbit pre-immune serum (i and vii), anti-FhStf-1 polyclonal antibodies (ii and viii), anti-FhStf-2 polyclonal antibodies (iii and ix), anti-FhStf-3 polyclonal antibodies (iv and x), anti-FhCL3 polyclonal antibodies (v and xi) or anti-FhCB3 polyclonal antibodies (vi and xii) followed by FITC-labelled secondary antibodies. FITC staining (green fluorescence) revealed FhStf-1, FhStf-2 and FhStf-3 proteins are located within the bifurcated gut at both time-points comparable to FhCL3 and FhCB3. All specimens were counter-stained with phalloidin-tetramethylrhodamine isothiocyanate (TRITC) to stain muscle tissue (red fluorescence) and provide structure. OS, oral sucker; VS, ventral sucker. Scale bars = 20 μM.

Recombinant proteins rFhStf-1, rFhStf-2, rFhStf-3 and rFhKT1 were purified by one-step Nickle-chelate affinity chromatography and resulted in proteins of high yield. All four recombinant proteins were analysed by SDS-PAGE and were shown to migrate as single bands at the expected molecular size (FhStefins ~11 kDa and FhKT1 ~6kDa) and be of high, >95%, purity (Figure 1A).

#### 3.1.2. The Recombinant *F. hepatica* Stefins and Kunitz-Type Inhibitor Are Potent Inhibitors of Parasite Cysteine Peptidases

Analysis of the inhibition profile of the recombinantly expressed parasite protease inhibitors (rFhStf-1, rFhStf-2, rFhStf-3 and FhKT1) demonstrate that they are functionally active and potent inhibitors of native *F. hepatica* cysteine peptidases found in both the NEJ and adult excretory/secretory products (ES). rFhStf-1 was shown to reduce the cysteine peptidase activity of NEJ ES (97% at 500 nM and 73.6% at 10 nM) and adult parasite ES (98.1% at 500 mM and 71.8% at 500 nM and 10 nM) (Figure 1B). Similar inhibition of NEJ and adult ES cysteine peptidase activity was also observed by rFhStf-2. In contrast, although rFhStf-3 showed similar inhibitions levels to the other stefins at 500 nM, reducing NEJ ES cysteine peptidase activity by 95% and adult ES activity by 98.8%, it exhibited noticeably lower levels of inhibition at 10 nM (NEJ ES: 8%; Adult ES: 49%; Figure 1B). We have previously reported that FhKT1 is also able to completely inhibit the cysteine peptidase activity within the adult ES products at concentrations greater than 250 nM [21]. Similarly, we observed that the inhibitors are also potent inhibitors of host cathepsin L peptidases (Table S1).

#### 3.1.3. Immunolocalisation Studies

Antibodies were prepared to each of the stefins, rFhStf-1, rFhStf-2, rFhStf-3, and used in immunolocalisation studies of fixed NEJ. All three inhibitors were located within the bifurcated gut, where the major parasite cysteine peptidases (FhCL3 and FhCB3) are also expressed (Figure 1C; [12]). Previous studies demonstrated that the FhKT1 was also expressed in the NEJ gut, as well as in narrow channels that penetrated the NEJ parenchymal tissue and sub-tegumental surface [22].

### 3.2. Vaccine Trials

#### 3.2.1. Assessment of Protection Based on Total Adult Fluke Enumeration and Egg Viability

Total adult fluke enumeration was carried out to determine the fluke burden for each group in both trials. In Trial 1, the fluke burdens were 17.4% lower in the vaccinated group (G3) compared to the control non-vaccinated group (G2) (Table 1). No protection was observed within the vaccinated groups of Trial 2 (G3 and G4) compared with the control non-vaccinated group (G2) (Table 1). The experimental *F. hepatica* isolate used for each vaccine trial was different (South Gloucester isolate for Trial 1 and Italian isolate for Trial 2) and displayed different viability/pathogenicity traits as shown by the number of flukes recovered relative to the infection dose. The size of the adult flukes obtained were also significantly different between the two trials (No. flukes *p* < 0.0001; Fluke length/width *p* < 0.05; Table 1; Table S3).

No significant differences were observed between the number of eggs assessed by FEC, with all groups in both trials shedding increasing numbers of eggs as the infection progressed (Table 2). In Trial 1, however, the number of eggs recovered from the gall bladder at post-mortem from the vaccinated animals (G3) was 46% lower compared to the number of eggs recovered from the non-vaccinated control group (G2) and these displayed a 36.2% reduction in viability based on the egg hatch results (Table 3 and Table 4). In Trial 2, no reduction in gall bladder eggs was observed for the vaccinated animals (G3) compared to the non-vaccinated control group (G2); however, a 43% reduction was observed in the vaccinated animals that also received the CpG oligonucleotide (G4). Furthermore, both vaccinated groups displayed a reduction in egg viability compared to the non-vaccinated infected control (G3: 38.5%; G4: 42.1%; Table 4).

#### 3.2.2. Haematological Analysis

**Figure 2:**
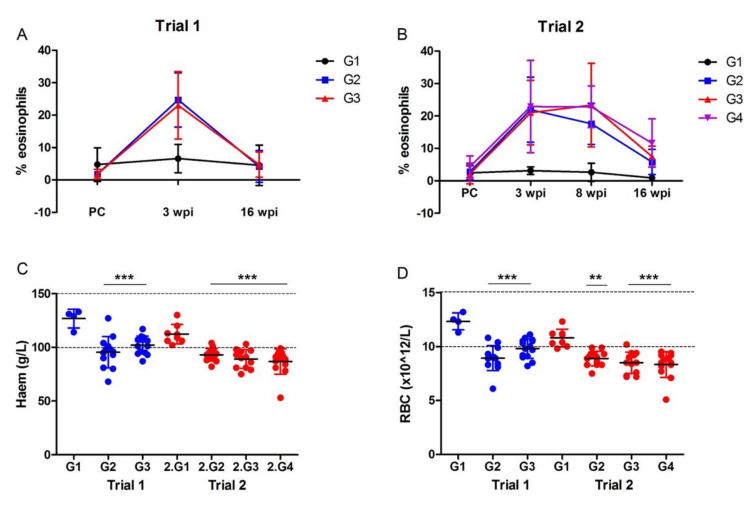
Profile of haematological parameters during *F. hepatica* infection. (**A**,**B**) Eosinophilia during the vaccine trial time-course represented as a percentage of the total cells. (**A**) In Trial 1, blood samples were taken post-vaccination/pre-challenge with *F. hepatica* metacercariae (PC), 3 wpi and 16 wpi. (**B**) In Trial 2, blood samples were taken post-vaccination/pre-challenge with *F. hepatica* metacercariae (PC), 3 wpi, 8 wpi and 16 wpi. Data represented at ± standard deviation. (**C**) Levels of haemoglobin measured as g/L in blood samples taken at 16 wpi from Trial 1 (blue) and Trial 2 (red), represented at ± standard deviation. Normal values typically range between 100 and 150 g/L as shown by the two lines. (**D**) Number of red blood cells (RBC; × 10^12^/L) in blood samples taken at 16 wpi from Trial 1 (blue) and Trial 2 (red), represented at ± standard deviation. Normal values typically range between 10 × 10^12^/L and 15 × 10^12^/L as shown by the two horizontal-dashed lines. G1, control unvaccinated, uninfected; G2, control unvaccinated, infected; G3 and G4, vaccinated, infected. ** *p* < 0.01 and *** *p* < 0.001 relative to the values obtained from the non-vaccinated, non-infected group (G1) in each trial.

In both vaccine trials, increased levels of eosinophilia were observed in all *F. hepatica* infected animals at 3 wpi compared to the start of the trials, with mean levels of ~20% of total cell counts (Figure 2). At 16 wpi, eosinophilia had reduced to levels comparable to the ~5% of total cells observed in the non-infected control group (G1). Vaccination had no effect on the levels of eosinophilia induced by infection, with no statistical differences observed between the vaccinated groups (G3/G4) and the control infected group (G2) in either trial. However, in Trial 2, the vaccinated groups displayed marginally higher mean percentage eosinophilia at 8 wpi and 16 wpi compared to the control infected group (8 wpi: G2, 17.6% ± 6.34; G3: 23.4% ± 12.9; G4: 22.8% ± 6.39. 16 wpi: G2, 5.82% ± 3.91; G3: 7.50% ± 3.17; G4: 11.6% ± 7.46).

At 16 wpi, the levels of haemoglobin and the RBC in the *F. hepatica* infected animals were significantly lower than the non-infected control animals (*p* < 0.001). In particular, most animals in Trial 2 had haemoglobin and RBC levels below the reference range (100–150 g/L) (Figure 2). These results correlated with the packed cell volume (PCV) data whereby animals infected with *F. hepatica* in Trial 2 (G2, G3, G4) displayed lower mean PCV values, with 95% confidence intervals of 0.27% [0.26, 0.28] compared with the non-infected controls (G1) 0.34% [0.30, 0.37]. Comparative analyses of the animals that received the same vaccine combination, namely Group 3 in each trial, showed that the haemoglobin and RBC levels were significantly higher in the animals in Trial 1 compared with Trial 2 (*p* < 0.05); this may indicate that the liver fluke isolates used in the trials induce different levels of pathogenicity.

#### 3.2.3. Liver Enzyme Assays

**Figure 3:**
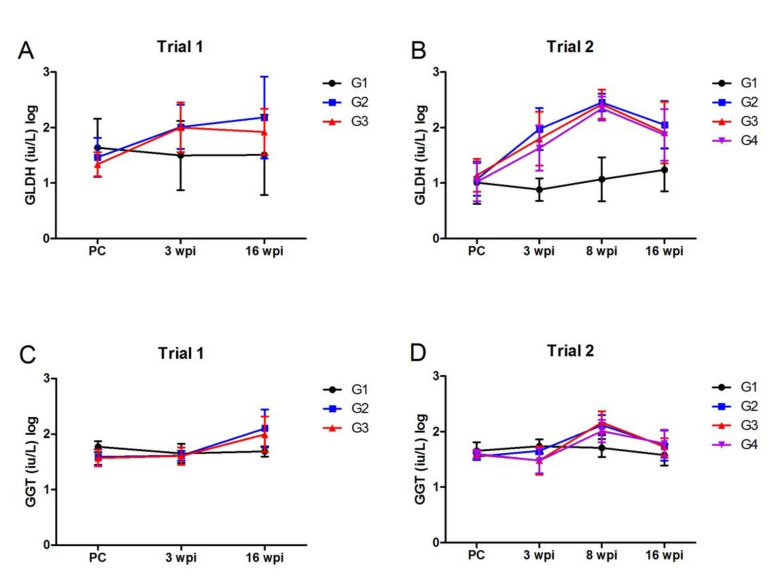
Profile of liver enzymes during *F. hepatica* infection. (**A**,**B**) Level of glutamate dehydrogenase (GLDH; units/L) in serum during the vaccine trial time-course presented on a log scale. (**A**) In Trial 1, serum samples were analysed at post-vaccination/pre-challenge with *F. hepatica* metacercariae (PC), 3 wpi and 16 wpi time-points. (**B**) In Trial 2, serum samples were analysed at post-vaccination/pre-challenge with *F. hepatica* metacercariae (PC), 3 wpi, 8 wpi and 16 wpi time-points. (**C**,**D**) Level of gamma glutamyl-transferase (GGT; units/L) in serum during the vaccine trial time-course presented on a log scale. (**C**) In Trial 1, serum samples were analysed at post-vaccination/pre-challenge with *F. hepatica* metacercariae (PC), 3 wpi and 16 wpi time-points. (**D**) In Trial 2, serum samples were analysed at post-vaccination/pre-challenge with *F. hepatica* metacercariae (PC), 3 wpi, 8 wpi and 16 wpi time-points. Data represented at ± standard deviation. G1, control unvaccinated, uninfected; G2, control unvaccinated, infected; G3 and G4, vaccinated, infected.

Serum levels of glutamate dehydrogenase (GLDH) increased following infection with *F. hepatica* in both trials, with no significant differences between the vaccinated (G3/G4) and the non-vaccinated control animals (G2). In Trial 2, the highest levels of serum GLDH were detected at 8 wpi (Figure 3). In comparison, the levels of gamma glutamyl transferase (GGT) did not increase substantially higher than those observed in the negative non-infected animals, with a marginal non-significant increase observed at 16 wpi in Trial 1 (Figure 3). Consistent with the serum GLDH levels, in Trial 2 the highest levels of serum GGT were also detected at 8 wpi. For both vaccine trials, no significant differences were observed between the vaccinated animals and the non-vaccinated control animals (G2).

#### 3.2.4. Weight Gain

**Figure 4:**
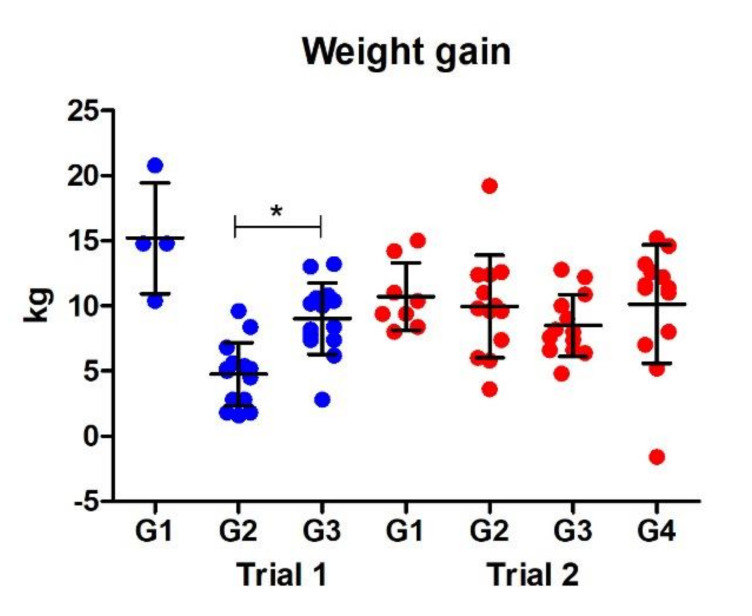
Graphical representation of the total weight gain during the vaccine trial. The weight gain for each animal was calculated as the difference between the final weight at necropsy and the starting weight at the beginning of the trial. Statistical difference between the control group (G2) and vaccinated group (G3) for Trial 1 is highlighted (* *p* < 0.05). G1, control unvaccinated, uninfected; G2, control unvaccinated, infected; G3 and G4, vaccinated, infected.

The weight of the animals was monitored throughout the study, and the final weight gain was calculated at post-mortem relative to the weights at the beginning of the vaccine trials (Figure 4). In Trial 1, animals in the vaccinated group (G3) showed a significantly greater weight gain (*p* < 0.05) relative to the non-vaccinated control group (G2; Figure 4); average weight gains in the two groups were 9.0 kg and 4.7 kg, respectively. The average weight in the vaccinated group (G3) showed no significant difference to the non-vaccinated, non-infected animals (G1). However, in Trial 2 no significant differences were observed between all groups, which displayed similar levels of weight gain of, on average, 9.7 kg.

### 3.3. Analysis of Immunogenicity of the Vaccine Antigens by Lymphocyte Stimulation Assays

**Figure 5:**
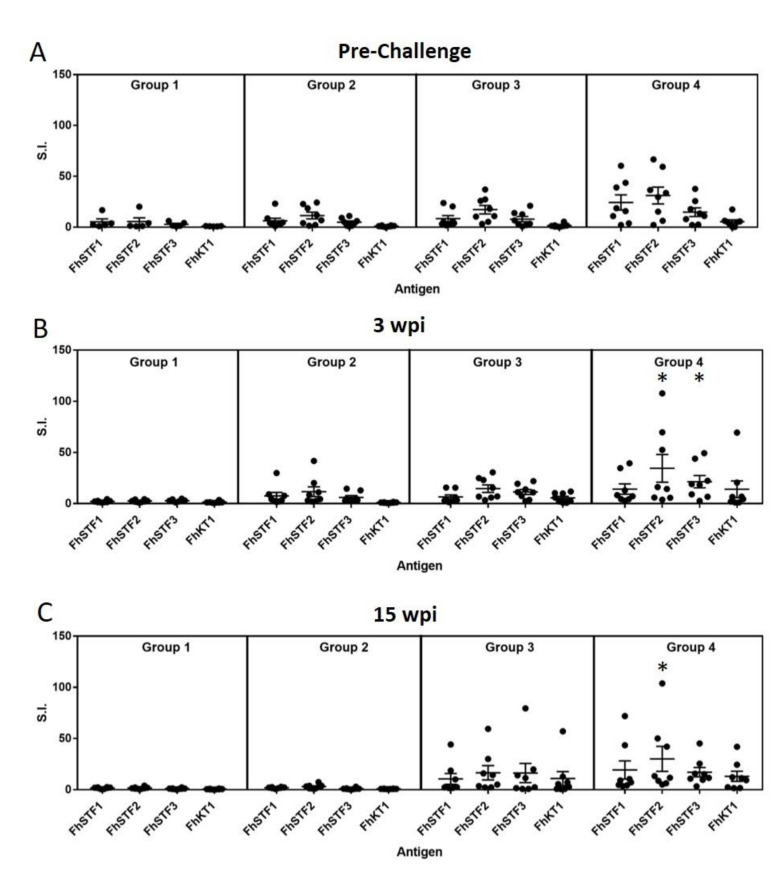
Proliferative responses of PBMC stimulated with the recombinant stefins and Kunitz-type antigens. PBMC were analysed at three time points, namely (**A**) prior to infection with *F. hepatica* metacercariae (pre-challenge), (**B**) 3 wpi and (**C**) 15 wpi. Proliferation is expressed as Stimulation Index (S.I.). Group 1, control unvaccinated, uninfected; Group 2, control unvaccinated, infected; Group 3 and Group 4, vaccinated, infected. * *p* < 0.05 relative to the values obtained from the non-vaccinated, non-infected group (G1) at the respective time-points.

In Trial 2, PBMC were isolated at three time-points from representative animals for each group, (i) after vaccination and before challenge with *F. hepatica* metacercariae, (ii) 3 wpi and (iii) 15 wpi, one week prior to necropsy. These were stimulated with the recombinant *F. hepatica* stefins and FhKT1, which were heat inactivated to prevent any bioactivity affecting the T-cell response (Figure 5). Comparative analysis of the stimulation index (S.I.) values revealed no statistical differences between the values obtained for the non-vaccinated, non-infected group (G1) compared with the infected animals (G2) at any time-point, indicating that the *F. hepatica* inhibitors do not stimulate an immune response during natural infection. A slightly broader range of S.I values were observed for the vaccinated groups (G3 and G4). The values for G3 were slightly higher, though not significantly different to those observed in the control groups (G1 and G2). Only the G4 vaccine group animals, that were vaccinated with the *F. hepatica* inhibitors and CpG, displayed significantly different values to the non-vaccinated, non-infected group (G1) for the PBMC stimulated with rFhStf2 and rFhStf3 at 3 wpi (*p* < 0.05) and rFhStf2 at 15 wpi (*p* < 0.05). These results indicate that the G3 vaccine induced a transient systemic cell-mediated immune response, but that this response waned over time, with more animals displaying values comparable to the control background levels by 15 wpi. In addition, although statistically significant results were obtained for the vaccinated animals in G4, this was not observed across all the four antigens in the vaccine cocktail. No correlation was observed for the level of stimulation compared with the number of flukes recovered at necropsy.

### 3.4. Analysis of Immunogenicity of the Vaccine Antigens by ELISA

**Figure 6:**
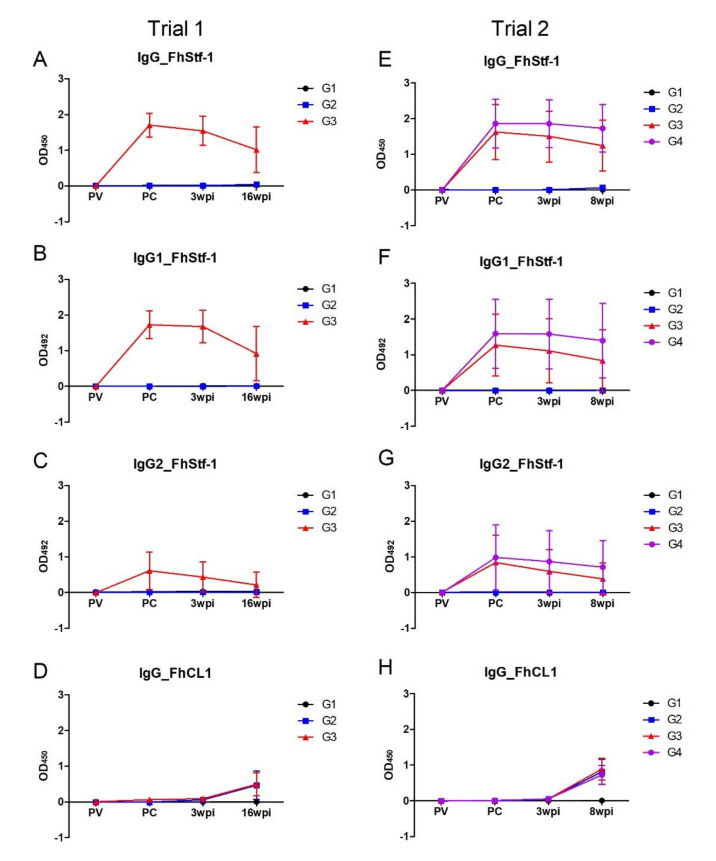
Analysis of antibody responses to *F. hepatica* recombinant stefin 1 (rFhStf-1). Mean optical density (OD) values are graphically represented for total IgG antibodies (**A** and **E**), IgG1 antibody subclass (**B** and **F**) and IgG2 antibody subclass (**C** and **G**) to rFhStf-1 at four time-points during the vaccine Trial 1 and Trial 2. Antibody responses to *F. hepatica* infection are displayed as mean OD values for total IgG antibodies to the recombinant cathepsin L peptidase (rFhCL1; **D** and **H**). PV, pre-vaccination, 0 wpi; PC, post-vaccination/pre-challenge with *F. hepatica* metacercariae. Data represented at ±standard deviation. G1, control unvaccinated, uninfected; G2, control unvaccinated, infected; G3 and G4, vaccinated, infected.

Antibody responses to the three recombinant *F. hepatica* stefins and Kunitz-type inhibitor vaccine antigens were determined at four time-points, namely at the beginning of the trial prior to vaccination (PV), after vaccination and before the challenge infection with *F. hepatica* (PC), at 3 wpi and at either 16 wpi (Trial 1) or 8 wpi (Trial 2). As a control, antibody responses to rFhCL1, a molecule not included in the vaccine but known as a highly immunogenic protein during infection with *F. hepatica* in ruminants [25], were also examined. Anti-rFhCL1 antibodies were detected in all *F. hepatica* infected animals following 3 wpi (Figure 6 and Figure S1). The four recombinant inhibitors did not elicit measurable antibody responses in the non-vaccinated infected control animals (G2) consistent with the PBMC proliferation assay that showed that stefins and FhKT1 are not immunogenic during natural infection (Figure 6 and Figure S1B–D).

Comparable results were observed for all four recombinant inhibitors, with significantly strong IgG antibody responses induced by the vaccination protocol in the vaccinated groups at the pre-challenge time point compared with the non-vaccinated animals (*p* < 0.001). However, these responses were not boosted following challenge infection and showed a decline as the infection progressed, though at the final time point analysed the OD values were higher than that observed for the immunogenic rFhCL1 (Figure 6 and Figure S2).

Analysis of the specific IgG1 and IgG2 subclass response showed a similar profile to the total IgG antibody response for all the four recombinant inhibitors, with particularly strong IgG1 antibody responses being observed (Figure 6 and Figure S2). IgG2 antibody responses were elicited by the vaccination protocol, however the OD values were considerably lower than that observed for IgG1. Comparative analysis of the two vaccine combinations in Trial 2 showed that the group that also received the CpG oligonucleotide (G4) had higher mean OD values corresponding to the total IgG and both IgG1 and IgG2 isotypes compared with the vaccine group 3 (G3), though due to the large variability between the animals this was not statistically significant (Figure 6 and Figure S2).

### 3.5. Correlation Analysis

The numerical variables generated by the trials were analysed for multicollinearity, which highlighted that the parameters associated with haemoglobin and RBC/PCV displayed high levels of correlation (correlation coefficient values between 0.78 and 0.89). Similarly, high levels of correlation were observed across the ELISA data (correlation coefficient values between 0.76–0.96). Therefore, haemoglobin levels and the ELISA data for FhKT1 antigen (IgG, IgG1, IgG2), were selected as representative variables of RBC phenotype and antibody response, respectively, for further analysis.

Multivariate regression analysis was carried out to determine if any variables from the vaccine trial data were correlated with positive outcomes of vaccination such as increased weight gain, haemoglobin levels within normal levels and reduced parasite burden. Weight gain, haemoglobin levels at 16 wpi and fluke number were used as dependent variables. The independent variables were selected following stepwise backward regression analysis representing a range of variables related to parasite date, vaccination, animal data, liver pathology, antibody responses and haematology parameters as shown in Table S4.

Whether or not animals were administered the liver fluke vaccine was a significant positive indicator for weight gain (*p* < 0.05). Increased levels of lymphocytes at 16 wpi and the parasite isolate used for infection were also associated with increased weight gain (*p* < 0.05). The low adjusted R2 value (20%) indicates that other variables not analysed in this study may be contributing to the resulting weight gain.

Liver fluke isolate was the only significant variable determining the number of parasites recovered at necropsy (*p* < 0.01) and was linked with the higher number of parasites recovered for the Italian isolate compared to the South Gloucester isolate.

Seven significant variables contributed to the haemoglobin levels at 16 wpi (R2 = 77%; *p* = 2.147 × 10^−12^). Increased levels of immune cells at 16 wpi, namely neutrophils, lymphocytes and eosinophils, and haemoglobin at 3 wpi were significantly correlated with haemoglobin within the normal range (*p* < 0.001). Animal vaccination and increased levels of IgG2 antibodies specific for the *F. hepatica* peptidase inhibitors were also positive indicators for increased levels of haemoglobin at 16 wpi (*p* < 0.05). Consistent with the observation that chronic fasciolosis is associated with increased levels of GGT, the regression modelling shows that lower levels of GGT at 16 wpi were associated with higher levels of haemoglobin, highlighting that the damage caused within the bile ducts is associated with the blood feeding activities of the parasite.

Key points from the multivariate regression modelling reveal the following correlations:

- Vaccination was associated with an average weight gain of 3.5 kg and increased haemoglobin levels of 7.18 g/L.
- The IgG2 antibodies elicited by vaccination were associated with average increased haemoglobin levels of 13.44 g/L.
- Infection with the Italian liver fluke isolate, which comprised of on average 42 more adult parasites than infections with the South Gloucester isolate, was associated with an average weight loss of 4.4 kg.

## 4. Discussion

The protease/anti-protease balance, described as the delicate interplay between enzymes and their inhibitors, finely regulates the activity of proteases across a range of living systems [31]. In the case of human disease, changing the dynamics between proteases and their inhibitors, leads to the progression of diseases such as cancer and emphysema [32,33]. For example, elevated expression of cathepsin cysteine peptidases and subsequent unregulated proteolysis has been implicated in a range of cancers [34]. The malignant progression of the cancerous cells and tumour formation is exacerbated by reduced levels of the endogenous inhibitors used to regulate the cathepsin peptidases, such as cystatin C that regulates cathepsin B in cases of tongue cancer [35] and breast cancer [36]. Moreover, decreased levels of Stefin A and Stefin B are associated with breast, prostate, cervical and brain cancers [37,38,39,40].

*F. hepatica* relies almost exclusively on a range of tightly regulated functionally diverse cathepsin peptidases that are temporally expressed and secreted throughout the parasite lifecycle [16]. Due to their importance for parasite biology, host invasion and feeding, the cathepsin peptidases have been the focus of several *F. hepatica* vaccine studies but have shown variable results [14,41]. Despite being highly immunogenic proteins in natural infection in sheep [25] the cathepsin peptidases do not seem to offer significant protection to re-infection [41]. Here we employed an alternative approach by targeting the inhibitors that regulate cathepsin peptidase activity in an attempt to disrupt the parasite protease/anti-protease balance. We hypothesised that if the protease/anti-protease balance was essential to the parasites’ ability to control their migration through tissues, to carefully regulate host immune responses and to assimilate nutrient from digested tissues/macromolecules then unsetting this rapport with its host by vaccination could elicit protection against infection.

The *F. hepatica* stefin and Kunitz-type inhibitors are derived from multi-copy gene families that display comparable differential expression throughout the life cycle to the cathepsin peptidase profile [12,13,21,23]. They are abundantly secreted by each developmental stage and have been identified on the outer surface of the juvenile parasite [12,13,21,23]. Their presence within the parasite secretory products is because of both classical secretion and encapsulation within extracellular vesicles (EVs). The *F. hepatica* cystatins are single domain cystatin-type inhibitors that are representative of the type 1 stefins I25A subgroup (MEROPS classification) that typically lack a secretory signal. While the FhStf1 follows this classical feature and is secreted by the parasite within EVs, FhStf2 and FhStf3 are unusual in possessing a signal peptide sequence that is more characteristic of the type 2 cystatins [23]. We have previously shown that the members of the secreted FhKT1 group are also secreted within the secretory products via classical secretory signals as well as being packaged within the EVs [21].

The potential for using these inhibitors for vaccine development is further enhanced by the fact that they are readily produced in large quantities using yeast expression systems. Our biochemical characterisation of the *F. hepatica* stefins and the *F. hepatica* Kunitz-type inhibitors shows that they are potent inhibitors of both parasite-secreted and host cysteine peptidases, suggesting a primary role in the regulation of parasite and host cysteine proteases that are involved in parasite processes such as invasion, development, feeding and immune evasion and host innate immune responses.

We assessed the efficacy of the *F. hepatica* inhibitor vaccine cocktail using protection parameters of parasite burden based on the number of adult parasites and faecal egg counts together with markers of fasciolosis disease in two independent sheep trials, which were carried out over two consecutive years. Characteristic features associated with fasciolosis were observed in both trials, namely an increase in the number of circulating eosinophils during infection from 3 wpi and the elevation of serum liver enzymes (GLDH and GGT) following liver damage caused by the migrating parasites. No significant differences were observed for the parasite burden based on the number of adult flukes recovered at necropsy and the faecal egg counts between the vaccinated and non-vaccinated animals in either trial. However, we did see a reduction in egg viability between the vaccinated and non-vaccinated animals in both trials, indicating that vaccination may have impacted the parasite’s blood feeding ability that is required for egg production. Other studies using native or recombinant cathepsins L have shown a significant impact on egg production/viability, even in the absence of reduced worm burden [41,42,43,44].

In both trials, a strong IgG antibody response was induced against the peptidase inhibitors that are normally not immunogenic during infection. This antibody response was comprised of both IgG1 and IgG2 subtypes, indicating a mixed Th1/Th2 response, comparable to that observed in other *F. hepatica* vaccine studies using the Montanide adjuvants [45,46]. In vaccine studies using Freund’s complete adjuvant, IgG2 responses correlated with lower parasites numbers, indicating that the induction of IgG2 responses during vaccination may be protective during *F. hepatica* infections [47]. In our studies, the IgG2 responses to all the peptidase inhibitors waned as infection progressed, which may have played a part in the low protective response of this particular antigen/adjuvant vaccine combination in terms of reducing the parasite burden. However, consistent with Mulcahy et al. [47], regression modelling highlights that increased levels of IgG2 antibodies specific for the *F. hepatica* peptidase inhibitors are indicators for the positive outcomes of vaccination such as increased weight gain and higher levels of haemoglobin at 16 wpi (*p* < 0.05).

Interestingly, we observed that, despite not conferring any protection, the addition of the CpG oligonucleotide to the vaccination cocktail in Trial 2 reduced the decline in IgG1 and IgG2 specific antibodies observed over the course of infection in comparison to those animals vaccinated with the antigens alone. At 3 wpi, IgG1 antibodies to the four antigens in group 4 (G4) remained at 98–100% of the mean OD values observed pre-challenge, in contrast to the means OD values of 58–95% observed for group 3 (G3). This observation was maintained at 8 wpi within the G4 group, with mean OD values ranging between 87 and 100% of the pre-challenge OD values compared with the G3 values (33–88%). Similarly, the IgG2-specific antibodies from G4 also followed this trend displaying mean OD values between 85–89% and 69–74% at 3 wpi and 8 wpi, respectively, compared with G3 mean OD values (3 wpi: 53–78%; 8 wpi: 28–56%). Significant differences were also observed in the lymphocyte stimulation assays for G4, indicating CpG enhanced Th1 responses in these animals, as reviewed by McCluskie et al. [48]. These results are consistent with other studies that have used CpG in their vaccine cocktail, which have shown prolonged humoral and cellular immune responses following vaccination [49]. Further investigation of the combinations of antigens, adjuvant and additives is therefore warranted for the development and optimisation of future *F. hepatica* vaccines.

An important positive outcome from Trial 1 was that the vaccinated animals displayed a significantly greater weight gain compared with the non-vaccinated control animals, which occurred without a significant reduction in parasite burden (17.4%). Zafra et al. [50] also reported in their cathepsin L1 vaccine study in goats significant weight gains in vaccinated animals despite no differences in liver fluke burden. Weight loss is a typical clinical sign associated with sub-acute disease resulting from poor feed-energy conversion, effecting the animal’s growth rate and overall weight and body condition, with reduced weight gain being a major effect of fluke infection [5]. Targeting the cathepsin peptidases by vaccination with the peptidases or peptidase inhibitors may have delayed the migration/progression of the parasite through the liver, resulting in less liver damage, reducing the pathogenicity associated with infection. This has the potential to minimise production losses, which is economically important, particularly to farmers [29,41].

We also evaluated vaccine efficacy in terms of a positive effect on animal productivity focusing on increased weight gain and markers of anaemia highlighted by the haemoglobin levels within a normal range. Regression analyses revealed that vaccination, which boosts IgG2 specific antibodies, together with increased levels of immune cells are associated with increased levels of haemoglobin and RBC/PCV, indicative of protection against blood loss and anaemia typically associated with acute infection. Similarly, vaccination and the parasite isolate were also significant positive indicators for weight gain, although other factors not assessed in this study/analyses are likely contributing to this effect.

Over the past two decades, *F. hepatica* vaccine studies have suffered from a lack of consistency and reproducibility [41]. Trials have been performed using different numbers of animals per group, using animals of different ages and varying numbers of parasites for challenge. In our studies, we used the same experimental design comprised of a large number of animals per group (13/14) calculated by power analysis, using the same breed and age of sheep in both trials, which were infected with the same parasite dose. The antigens were recombinantly expressed using standardised protocols and formulated in the same water–oil emulsion Montanide 61VG adjuvant. In both trials, we observed a wide range of variation across all the parameters used to assess vaccine efficacy, highlighting the need for large numbers of animals per group to determine significant and robust results in these types of trials. Despite observing positive results in Trial 1, these were not repeated in the second trial using a different set of animals, further stressing the animal-to-animal variation observed during *F. hepatica* vaccine trials.

Of note is the fact that we recovered more parasites, which were of a larger size from those animals infected with the Italian isolate in Trial 2 compared to the South Gloucester isolate used in Trial 1. Furthermore, more animals infected with the Italian isolate displayed levels of haemoglobin below the normal range that could be associated with anaemia, than those infected within the South Gloucester isolate, indicating that the number and size of the Italian isolate parasites may have induced more damage during the chronic stages of infection. This highlights possible significant variation between parasite isolates of different sources in terms of their potential pathogenicity and batch-to-batch fitness, which needs to be taken into consideration during *F. hepatica* experimental vaccine trials.

Another relevant difference between the two trials concerns the sex of the animals. The first trial was conducted using predominantly female sheep, while the second trial used male sheep. However, regression modelling indicated that sex was not a significant factor in terms of positive outcomes of vaccination such as increased weight gain and haemoglobin levels within normal levels. Male lambs typically display faster growth rates and attain a greater mature weight than female lambs [51,52,53]. In particular, Texel sheep demonstrate greater growth and muscularity in comparison to other sheep breeds [54] and mixed Texel-breeds show better growth performance [55]. In the context of fasciolosis infections, our analysis is consistent with the meta-analyses performed by Hayward et al. [5] who concluded that sex of the animal had no influence on weight gain parameters, whereas the age of the animal played an important role in the outcome and severity of disease and the impact on animal productivity. A preliminary study by Wesołowska et al. [44] reported that host sex influenced the outcome of infection following vaccination, with female calves and male lambs showing better protection than their counterparts, using group sizes of six animals per group. In contrast, our studies show that the more positive outcomes of vaccination were observed in the female lambs of Trial 1, although no major conclusions can be made regarding the influence of sex on vaccine efficacy from this current data. This highlights the need for more consideration and analysis in this area.

## 5. Conclusions

Previously, we and others have attempted to protect sheep and cattle against fasciolosis by targeting the parasite cathepsin cysteine peptidases via vaccination programs using either native or recombinant proteins, with variable success [41]. Here, we similarly targeted the cathepsin peptidases, taking a different approach focusing on a cocktail of cathepsin peptidase inhibitors with the expectation of upsetting the protease/anti-protease balance. However, while we observed some significant impacts on weight gain and egg production/viability these were nowhere near the threshold needed for an on-farm vaccine. Nevertheless, in our search for anti-liver fluke vaccines to replace or reduce the use of chemical treatments, we are learning more about the vagaries of large animal vaccine trials including the variabilities between parasite isolates, the need to use statistically robust numbers of animals per group, the absolute requirement for repeat trials and better understanding of the parameters of vaccine assessment. In the latter case, we need to further examine whether the common means of vaccine assessment of fluke burden is wholly appropriate or are other factors such as weight gain and haemoglobin concentration better indicators of animal health. The accumulation of vaccine trial data and the definition of these key outcome variables will allow for improved sample size calculations in subsequent trials. Finally, it is imperative that we continue to perform trials with new antigens/antigen cocktails in relevant target hosts to further refine the best candidates for vaccine development and continue to evaluate novel adjuvants and delivery systems for fluke vaccination.

## Figures and Tables

**Table 1:** Number and mean size of adult liver fluke recovered at necropsy.

Trial	Group	Mean Fluke Burden ± SD	Range	Protection (%)	Length (cm) ± SD
1	G2	60.71 ± 12.43	38–78	-	1.71 ± 0.108
1	G3	50.14 ± 16.27	29–81	17.4	1.62 ± 0.260
2	G2	87.23 ± 24.63	49–143	-	2.26 ± 0.467
2	G3	102.10 ± 24.32	65–145	0	2.14 ± 0.312
2	G4	86.92 ± 22.86	46–124	0	2.12 ± 0.166

**Table 2:** Faecal egg count (FEC) represented as egg per gram (EPG).

		Mean EPG ± SD		
Trial	Group	12 wpi	14 wpi	16 wpi
1	G2	33.0 ± 17.6	38.3 ± 15.9	62.3 ± 30.7
1	G3	32.4 ± 19.6	39.4 ± 20.9	61.4 ± 49.6
2	G2	27.3 ± 13.0	56.7 ± 25.9	55.4 ± 26.8
2	G3	24.4 ± 14.7	56.1 ± 40.5	64.4 ± 38.3
2	G4	57.1 ± 30.2	60.0 ± 33.9	59.6 ± 26.6

**Table 3:** Eggs recovered from gall bladder at necropsy.

Trial	Group	Number of Eggs ± SD	Reduction (%) *
1	G2	2.06 × 10^5^ ± 2.08 × 10^5^	-
1	G3	1.12 × 10^5^ ± 1.17 × 10^5^	46
2	G2	1.01 × 10^6^ ± 1.15 × 10^6^	-
2	G3	1.08 × 10^6^ ± 1.07 × 10^6^	0
2	G4	0.58 × 10^6^ ± 0.47 × 10^6^	43

* Relative to the number of eggs recovered from G2 control infected animals in each trial.

**Table 4:** Relative embryonation and hatch rate of eggs recovered from the gall bladder at necropsy.

Trial	Group	Embryonation (%)	Hatch Rate (%)	Protection (%) *
1	G2	52.7 ± 22.9	29.0 ± 18.7	-
1	G3	49.1 ± 23.9	18.5 ± 12.2	36.2
2	G2	58.8 ± 16.3	33.5 ± 13.0	-
2	G3	60.0 ± 18.9	20.6 ± 10.8	38.5
2	G4	67.2 ± 11.0	19.4 ± 6.8	42.1

* Based on mean hatch rate relative to the egg hatch rate of eggs recovered from G2 control infected animals in each trial.

## Data Availability

All data generated or analysed during this study are included in this published article and its Supplementary Materials files.

## Supplementary Materials

The following supporting information can be downloaded at: https://www.mdpi.com/article/10.3390/vaccines10020155/s1, Figure S1: Evaluating the level of cross reactivity between the *Fasciola hepatica* stefin antibodies. Figure S2: Analysis of antibody responses to the *F. hepatica* recombinant antigens used in the vaccine cocktail compared with the diagnostic antigen, cathepsin L peptidase displayed as per Figure 6. Table S1: Inhibition assays to determine specificity of the recombinant protease inhibitors (rFhStf-1, rFhStf-2, rFhStf-3 and rFhKT1). Table S2: Animal groups. Table S3: Mean size of adult flukes recovered at necropsy. Table S4: Parameters used for the multivariate regression analysis and details of the respective results.
