# Supplementary material for: Targeting Secreted Protease/Anti-Protease Balance as a Vaccine Strategy against the Helminth Fasciola hepatica
Source: Vaccines (Basel). 2022 Jan 20;10(2):155. doi: 10.3390/vaccines10020155 (PMC8878381; doi:10.3390/vaccines10020155)
Supplement: Supplementary file 1 [file vaccines-10-00155-s001.zip › vaccines-1494525-supplementary.pdf]

Supplementary Materials

Figure S1. Evaluating the level of cross reactivity between the *Fasciola hepatica* stefin antibodies.

Methodology: rFhStf-1, FhStf-2 and FhStf-3 (2µg) were added to equal volumes of Laemmli sample buffer (BioRad) with 50 mM DTT, heated at 90°C for 10 min. Gel electrophoresis was carried out using 4-20% Mini-PROTEAN® TGX precast protein gel (BioRad) 1 x SDS running buffer at 115 V for 1h and 10 min.), with the Precision Plus Protein™ Dual Xtra pre-stained protein standard (BioRad). The proteins were transferred to nitrocellulose membranes in semi-dry conditions for 15 min at 25 V using the Trans-blot Turbo transfer system (BioRad). The membranes were blocked for 1h at room temperature in phosphate-buffered saline with 0.05% Tween 20 Detergent (PBST) with 5% skimmed milk, followed by three five min washes in PBST. The membranes were then probed with primary antibody (pre-immune sera, αFhStf-1, αFhStf-2, αFhStf-3) diluted 1:150,000 in PBST with 2.5% milk for 1 h at room temperature. Following three five min washes in PBST, the membranes were probed with the secondary antibody, goat anti-rabbit IgG alkaline phosphatase conjugated (Sigma Aldrich) diluted 1:10,000 in PBST with 2.5% milk, for 1 h at room temperature. After three further five min washes in PBST, the membranes were developed for 2 min 30s with SigmaFast BCIP/NBT (Sigma Aldrich) and imaged using the G:BOX Chemi XRQ imaging system.

Western blot analysis of the three *F. hepatica* recombinant stefins probed with the polyclonal *F. hepatica* stefin antibodies. Immunoblots were probed with rabbit pre-immune serum as a negative control (Pre-immune), anti-rFhStf1 polyclonal antibodies raised in rabbit (αFhStf-1), anti-rFhStf2 polyclonal antibodies raised in rabbit (αFhStf-2), anti-rFhStf3 polyclonal antibodies raised in rabbit (αFhStf-3).

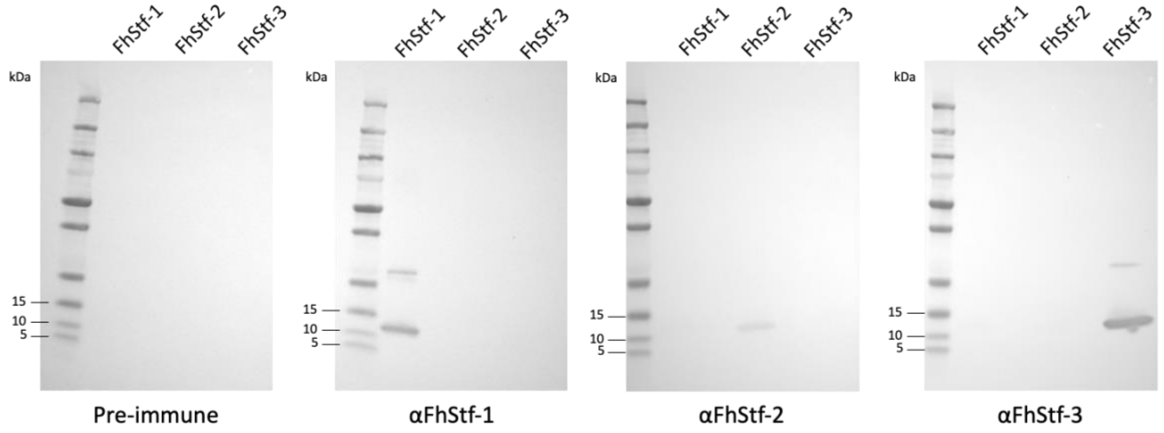

Figure S2. Analysis of antibody responses to the *F. hepatica* recombinant antigens used in the vaccine cocktail compared with the diagnostic antigen, cathepsin L peptidase, displayed as per Figure 6.

(A) Antibody responses to the *F. hepatica* recombinant cathepsin L1 peptidase (rFhCL1).

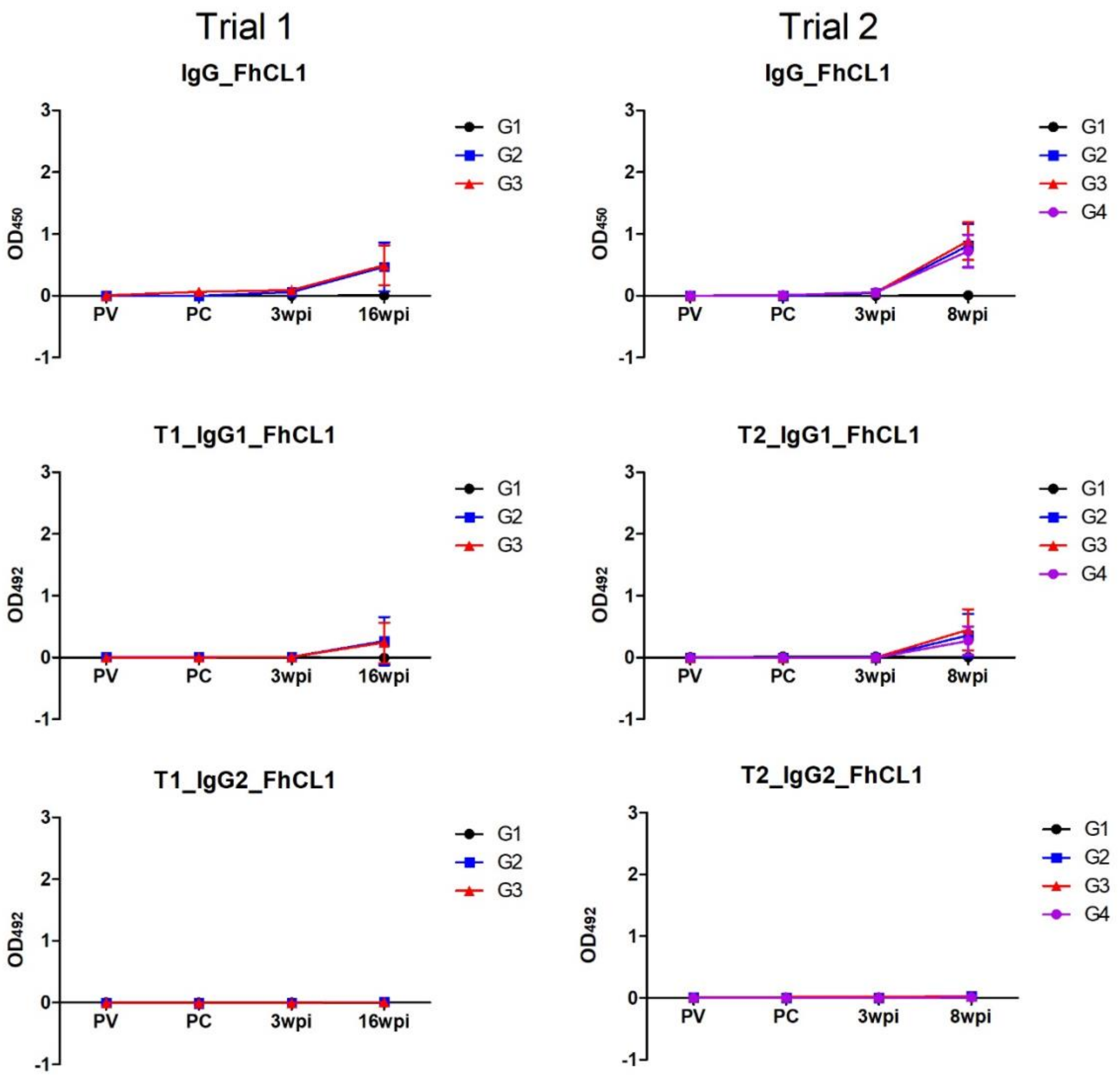

(B) Antibody responses to *F. hepatica* recombinant Stefin 2 (rFhStf-2).

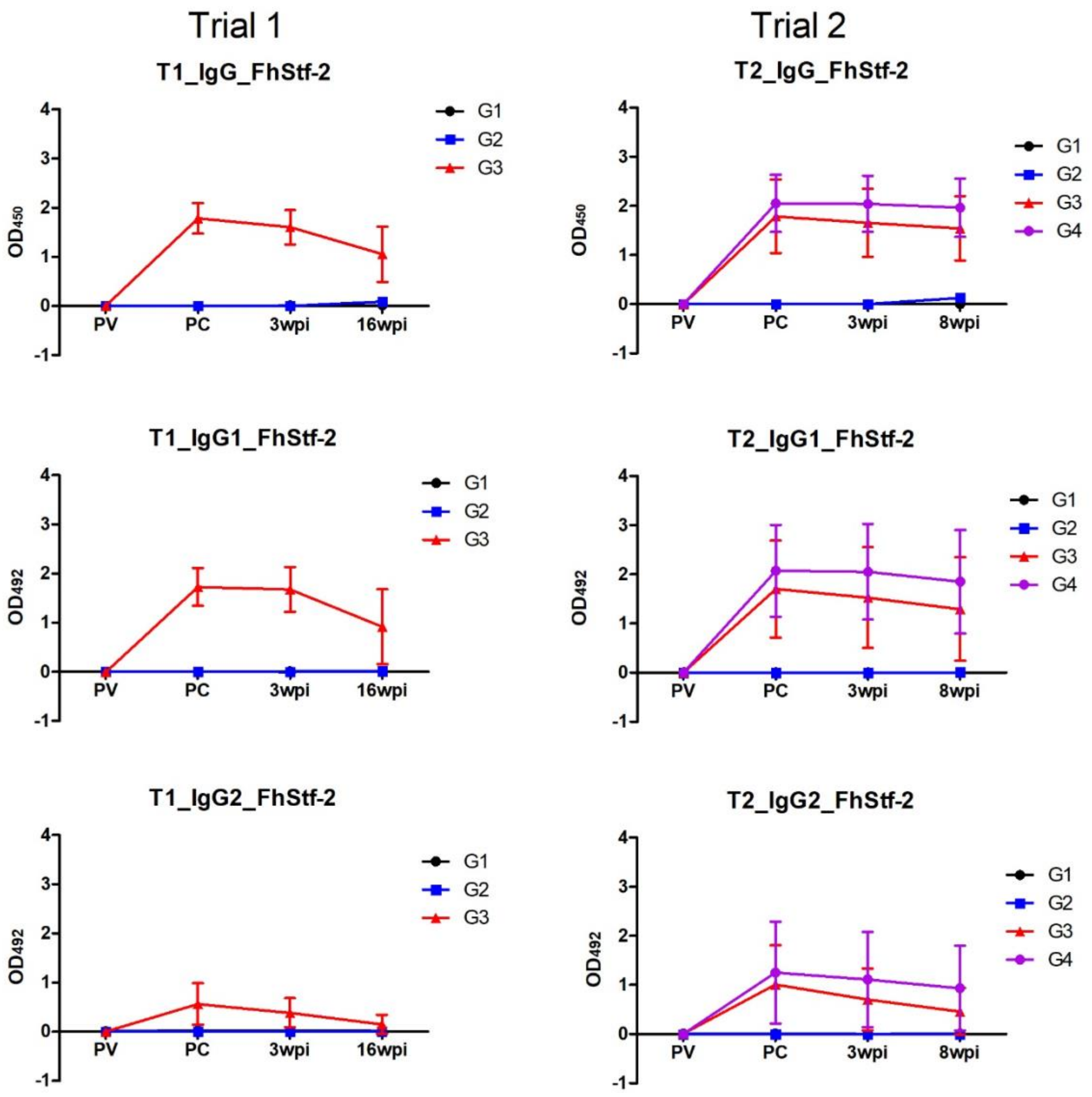

(C) Antibody responses to *F. hepatica* recombinant Stefin 3 (rFhStf-3).

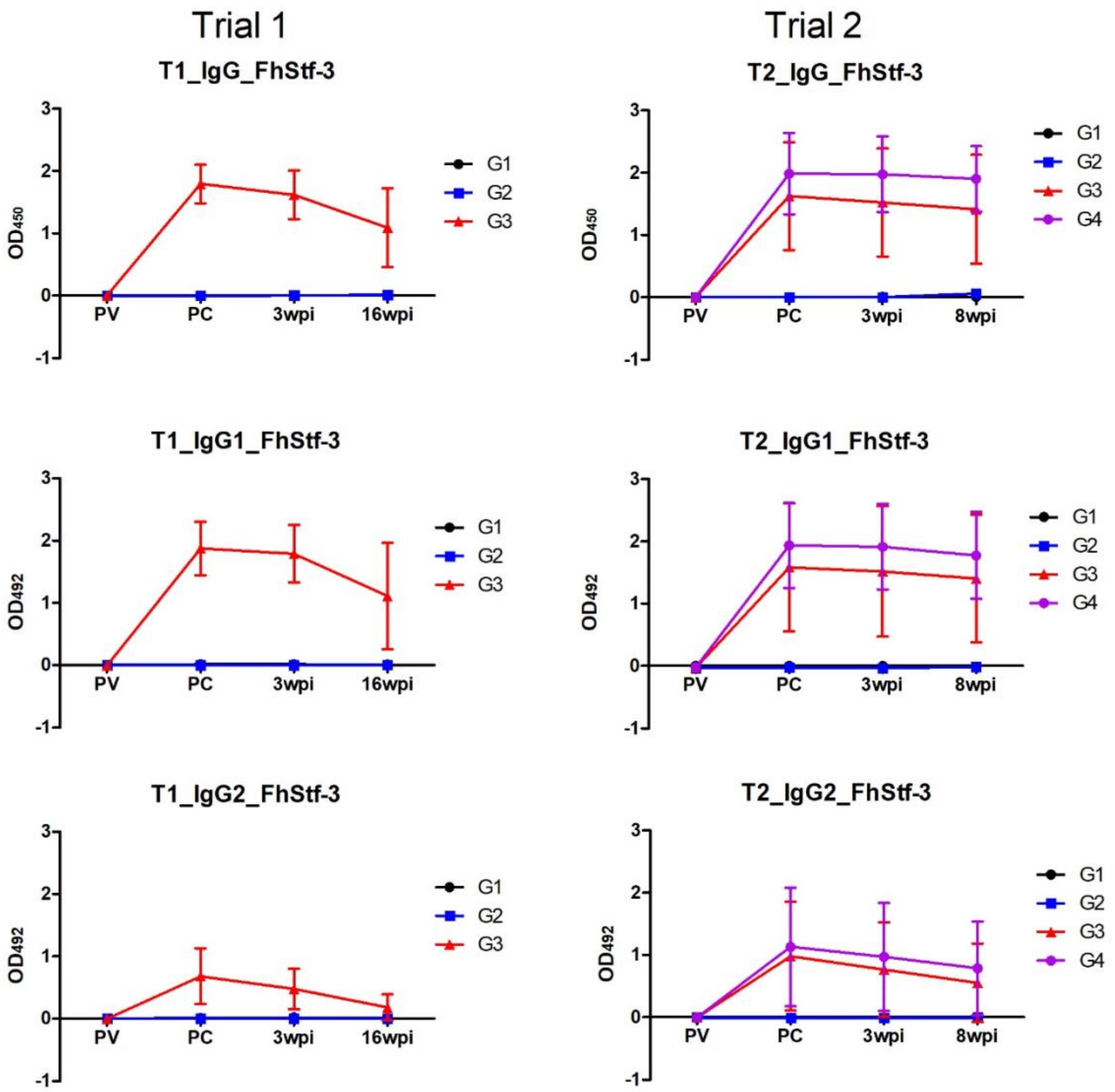

(D) Antibody responses to *F. hepatica* recombinant Kunitz-type inhibitor 1 (rFhKT1).

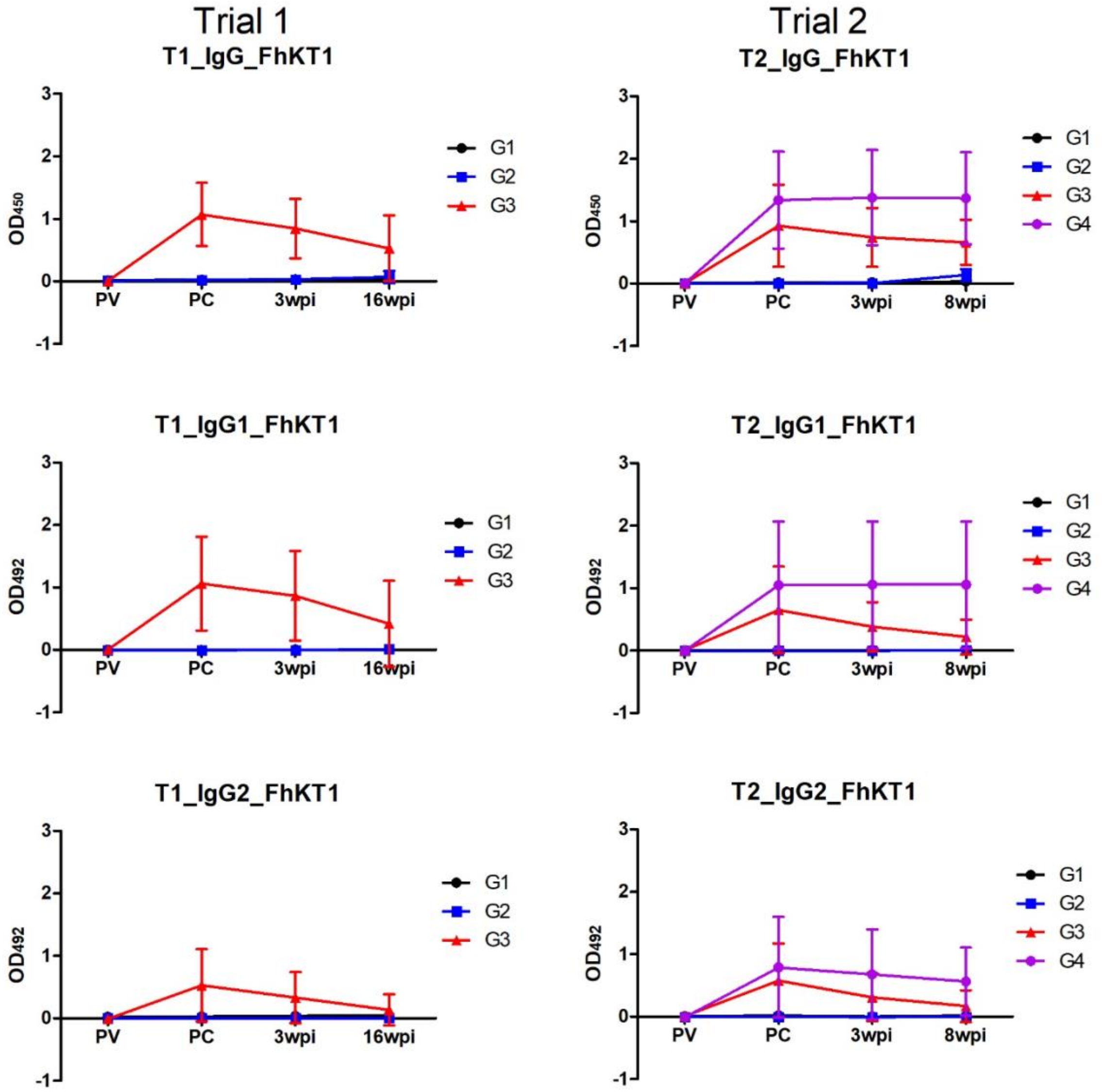

Table S1. Inhibition assays to determine specificity of the recombinant protease inhibitors (rFhStf-1, rFhStf-2, rFhStf-3 and rFhKT1).

Methodology: The inhibitory specificity of the recombinant proteins was analysed against a range of *F. hepatica* and mammalian cysteine proteases. rFhStf-1, rFhStf-2 and rFhStf-3 (500 nM and 10 nM) were first incubated with each protease in 100 µl of reaction buffer for 10 min at 37°C. The reaction volumes were brought to 200 µl with the addition of fluorogenic substrate dissolved in reaction buffer. Proteolytic activity was measured as RFU (relative fluorescent units) using a PolarStar Omega spectrophotometer (BMG LabTech, UK). Inhibition constants were determined using the Morrison equation for tight-binding inhibition as previously described [21]. All the screening assays were performed in triplicate at 37°C, using the reaction conditions and substrates detailed below (A). The *F. hepatica* cysteine proteases, cathepsin L1 (rFhCL1), cathepsin L2 (rFhCL2) and cathepsin L3 (rFhCL3) were expressed as functionally active recombinant proteins in *P. pastoris* as previously described [23]. The human cysteine proteases used in this study included human cathepsin B, cathepsin L and cathepsin S (All from Sigma-Aldrich) and human cathepsin K (Enzo Life Sciences). The screening assays performed using the Kunitz-type inhibitor, rFhKT1, were previously described by Smith et al. [21,22].

A. Flurogenic assay conditions for protease inhibition screening.

Enzyme	Assay Buffer*	Substrate (20 µM)
F. hepatica cathepsin rL1 (rFhCL1; 2.7 nM)	50 mM Citrate Phosphate Buffer	Z-Leu-Arg-NHMec
F. hepatica cathepsin rL2 (rFhCL2; 5 nM)	50 mM Citrate Phosphate Buffer	Z-Leu-Arg-NHMec
F. hepatica cathepsin rL3 (rFhCL3; 5 nM)	100 mM Sodium Acetate Buffer	Z-Gly-Pro-Arg-NHMec

F. hepatica NEJ ES	100 mM Sodium Acetate Buffer	Z-Gly-Pro-Arg-NHMec
F. hepatica Adult ES	100 mM Sodium Acetate Buffer	Z-Leu-Arg-NHMec
Human cathepsin L (HsCL; 0.2 nM)	100 mM Sodium Acetate Buffer	Z-Phe-Arg-NHMec
Human cathepsin K (HsCK; 2 nM)	100 mM Sodium Acetate Buffer	Z-Phe-Arg-NHMec
Human cathepsin S (HsCS; 2 nM)	100 mM Sodium Acetate Buffer	Z-Val-Val-Arg-NHMec
Human cathepsin B (HsCB; 3.6 nM)	100 mM Sodium Acetate Buffer	Z-Phe-Arg-NHMec

*All assay buffers were pH 5.5 and contained 1 mM DTT, 1 mM EDTA, 0.01% Brij L23

B. Relative inhibition constants (K_i) for rFhStf-1, rFhStf-2, rFhStf-3 and rFhKT1 against a panel of biologically relevant cysteine proteases.

	Inhibition Constant (K_i), in nM			
Enzyme	rFhStf-1	rFhStf-2	rFhStf-3	rFhKT1
F. hepatica cathepsin peptidases				
rFhCL1	0.9 (± 0.04)	0.09 (± 0.03)	0.3 (± 0.01)	0.4 (± 0.1)*
rFhCL2	0.2 (± 0.02)	0.1 (± 0.02)	6.2 (± 2.1)	10 (± 0.3)*
rFhCL3	0.2 (± 0.08)	0.1 (± 0.01)	16 (± 3.2)	1.8 (± 0.6)#
Human cathepsin peptidases				
Cathepsin L	0.4 (± 0.06)	0.02 pM (± 0.03 pM)	1.8 (± 0.3)	1.6 (± 0.1)*
Cathepsin K	0.5 (± 0.2)	0.08 (± 0.07)	1 (± 0.1)	5 (± 0.3)*
Cathepsin S	2 (± 0.02)	0.02 (± 0.009)	2 (± 0.05)	NI*
Cathepsin B	6.6 (± 1.4)	0.2 (± 0.02)	45 (± 0.9)	NI*

NI: Not Inhibited
*As reported by Smith et al. [21]
#As reported by Smith et al. [20]

Table S2. Animal groups.

Trial	Group	Description
1	G1	Non-vaccinated, non-infected control
1	G2	Infected control
1	G3	rFhStf-1, rFhStf-2, rFhStf-3 & rFhKT1 plus Montanide ISA 61 VG
2	G1	Non-vaccinated, non-infected control
2	G2	Infected control
2	G3	rFhStf-1, rFhStf-2, rFhStf-3 & rFhKT1 plus Montanide ISA 61 VG
2	G4	rFhStf-1, rFhStf-2, rFhStf-3 & rFhKT1 plus CpG and Montanide ISA 61 VG

Table S3. Mean size of adult flukes recovered at necropsy.

Trial	Group	Length (cm) ±SD	Width (cm) ±SD	Area (cm^2) ±SD
1	G2	1.71 ± 0.108	0.599 ± 0.081	ND
1	G3	1.62 ± 0.260	0.581 ± 0.133	ND
2	G2	2.26 ± 0.467	0.781 ± 0.131	1.60 ± 0.620
2	G3	2.14 ± 0.312	0.817 ± 0.105	1.50 ± 0.388
2	G4	2.12 ± 0.166	0.785 ± 0.108	1.42 ± 0.306

ND – not determined

Table S4. Parameters used for the Multivariate Regression analysis and details of the respective results.

A. Variables displaying correlation coefficients of ≤0.8	
Parasite data	***Liver pathology parameters***
Adult fluke number	Liver pathology score*
Parasite isolate	GLDH levels at 3 wpi
Number of eggs in gall bladder	GLDH levels at 16 wpi*
Mean length of adult fluke	GGT levels at 3 wpi
	GGT levels at 16 wpi
Vaccine information	
Vaccine administration	***Haematological parameters***
CpG included formulation	% Neutrophils at 3 wpi
	% Neutrophils at 16 wpi
Animal	% Lymphocytes at 3 wpi
Sex	% Lymphocytes at 16 wpi
Weight gain	% Monocytes at 3 wpi
	% Monocytes at 16 wpi
ELISA data	% Eosinophils at 3 wpi
Anti-FhKT1 IgG at 3 wpi	% Eosinophils at 16 wpi
Anti-FhKT1 IgG1 at 3 wpi	Haemoglobin levels at 3 wpi
Anti-FhKT1 IgG2 at 3 wpi	Haemoglobin levels at 16 wpi

* Removed after stepwise backward regression analysis. wpi: weeks post-infection.

**B. Weight gain model**	Estimate	Std. Error	t value	Pr(>|t|)
(Intercept)	-20.718262	48.784206	-0.425	0.6730
Sex-Male	-2.606739	1.948522	-1.338	0.1875
Vaccine-Yes	3.501933	1.516680	2.309	0.0255 *
Isolate-South Gloucester	-4.388211	2.156797	-2.035	0.0477 *
CpG- Yes	0.719172	1.600001	0.449	0.6552
GLDH_3wpi	-0.006395	0.005320	-1.202	0.2354
GGT_3wpi	0.066568	0.038613	1.724	0.0914 .
GGT_16wpi	-0.001814	0.005141	-0.353	0.7259
Neutrophils_3wpi	0.092877	0.469650	0.198	0.8441
Neutrophils_16wpi	0.046092	0.057392	0.803	0.4260
Lymphocyte_3wpi	0.098554	0.460899	0.214	0.8316
Lymphocyte_16wpi	0.101266	0.047153	2.148	0.0370 *
Monocyte_3wpi	0.544824	0.583441	0.934	0.3553
Monocyte_16wpi	-0.301409	0.357789	-0.842	0.4039
Eosinophils_3wpi	0.127782	0.460107	0.278	0.7825
Eosinophils_16wpi	0.132751	0.118791	1.118	0.2696
Haem_3wpi	0.099892	0.082996	1.204	0.2349
IgG_FhKT1_3wpi	-3.667444	3.250359	-1.128	0.2650
IgG1_FhKT1_3wpi	-0.479227	2.421781	-0.198	0.8440
IgG2_FhKT1_3wpi	3.342758	2.593903	1.289	0.2039

Signif. codes: 0 ‘***’, 0.001 ‘**’, 0.01 ‘*’, 0.05 ‘.’, 0.1 ‘ ’, 1
Residual standard error: 3.384 on 46 degrees of freedom
Multiple R-squared: 0.4392, Adjusted R-squared: 0.2075
F-statistic: 1.896 on 19 and 46 DF, p-value: 0.03912

C. Haemoglobin at 16 wpi model

	Estimate	Std. Error	t value	Pr(>|t|)
(Intercept)	-44.793127	110.932891	-0.404	0.6882
Sex-Male	-0.232692	4.430844	-0.053	0.9583
Vaccine-Yes	7.181043	3.448857	2.082	0.0429 *
Isolate-South Gloucester	8.454770	4.904450	1.724	0.0914 .
CpG- Yes	-3.416858	3.638324	-0.939	0.3526
GLDH_3wpi	-0.005457	0.012097	-0.451	0.6540
GGT_3wpi	0.086757	0.087804	0.988	0.3283
GGT_16wpi	-0.027920	0.011691	-2.388	0.0211 *
Neutrophils_3wpi	-1.195753	1.067961	-1.120	0.2687
Neutrophils_16wpi	1.181741	0.130507	9.055	8.67e-12 ***
Lymphocyte_3wpi	-1.070384	1.048062	-1.021	0.3125
Lymphocyte_16wpi	1.332453	0.107224	12.427	2.63e-16 ***
Monocyte_3wpi	-0.253090	1.326716	-0.191	0.8495
Monocyte_16wpi	1.273821	0.813596	1.566	0.1243
Eosinophils_3wpi	-0.737688	1.046261	-0.705	0.4843
Eosinophils_16wpi	1.481505	0.270126	5.485	1.70e-06 ***
Haem_3wpi	0.979771	0.188728	5.191	4.61e-06 ***
IgG_FhKT1_3wpi	-13.843162	7.391156	-1.873	0.0674 .
IgG1_FhKT1_3wpi	0.251332	5.507012	0.046	0.9638
IgG2_FhKT1_3wpi	13.441534	5.898407	2.279	0.0274 *

Signif. codes: 0 ‘***’, 0.001 ‘**’, 0.01 ‘*’, 0.05 ‘.’, 0.1 ‘ ’, 1
Residual standard error: 7.696 on 46 degrees of freedom
Multiple R-squared: 0.8387, Adjusted R-squared: 0.7721
F-statistic: 12.59 on 19 and 46 DF, p-value: 2.147e-12

D. Fluke number model

	Estimate	Std. Error	t value	Pr(>|t|)
(Intercept)	324.815025	302.455497	1.074	0.28846
Sex-Male	-7.678076	12.080576	-0.636	0.52820
Vaccine-Yes	6.206703	9.403214	0.660	0.51251
Isolate-South Gloucester	-42.662987	13.371850	-3.191	0.00256 **
CpG-Yes	-1.023274	9.919790	-0.103	0.91829
GLDH_3wpi	0.060903	0.032981	1.847	0.07124 .
GGT_3wpi	-0.414985	0.239394	-1.733	0.08971 .
GGT_16wpi	0.006342	0.031876	0.199	0.84318
Neutrophils_3wpi	-1.077116	2.911768	-0.370	0.71314
Neutrophils_16wpi	-0.320339	0.355824	-0.900	0.37266
Lymphocyte_3wpi	-0.836674	2.857511	-0.293	0.77099
Lymphocyte_16wpi	-0.588364	0.292343	-2.013	0.05003 .
Monocyte_3wpi	-3.771954	3.617254	-1.043	0.30251
Monocyte_16wpi	-2.158900	2.218246	-0.973	0.33552
Eosinophils_3wpi	-1.375086	2.852601	-0.482	0.63206
Eosinophils_16wpi	-0.730712	0.736490	-0.992	0.32631
Haem_3wpi	-0.531728	0.514562	-1.033	0.30684
IgG_FhKT1_3wpi	-8.409515	20.151785	-0.417	0.67839
IgG1_FhKT1_3wpi	4.287234	15.014718	0.286	0.77652
IgG2_FhKT1_3wpi	0.015487	16.081846	0.001	0.99924

Signif. codes: 0 ‘***’, 0.001 ‘**’, 0.01 ‘*’, 0.05 ‘.’, 0.1 ‘ ’, 1
Residual standard error: 20.98 on 46 degrees of freedom
Multiple R-squared: 0.5523, Adjusted R-squared: 0.3673
F-statistic: 2.986 on 19 and 46 DF, p-value: 0.001248
